# A2A Adenosine Receptor Antagonism Enhances Synaptic and Motor Effects of Cocaine via CB1 Cannabinoid Receptor Activation

**DOI:** 10.1371/journal.pone.0038312

**Published:** 2012-06-08

**Authors:** Alessandro Tozzi, Antonio de Iure, Valentina Marsili, Rosaria Romano, Michela Tantucci, Massimiliano Di Filippo, Cinzia Costa, Francesco Napolitano, Nicola Biagio Mercuri, Franco Borsini, Carmen Giampà, Francesca Romana Fusco, Barbara Picconi, Alessandro Usiello, Paolo Calabresi

**Affiliations:** 1 Clinica Neurologica, Università di Perugia, Ospedale S. Maria della Misericordia, Perugia, Italy; 2 Fondazione Santa Lucia - IRCCS, Rome, Italy; 3 CEINGE - Biotecnologie Avanzate, Naples, Italy; 4 Sigma - tau Industrie Riunite, Pomezia, Italy; 5 Second University of Naples (SUN), Naples, Italy; Institut National de la Santé et de la Recherche Médicale, France

## Abstract

**Background:**

Cocaine increases the level of endogenous dopamine (DA) in the striatum by blocking the DA transporter. Endogenous DA modulates glutamatergic inputs to striatal neurons and this modulation influences motor activity. Since D2 DA and A2A-adenosine receptors (A2A-Rs) have antagonistic effects on striatal neurons, drugs targeting adenosine receptors such as caffeine-like compounds, could enhance psychomotor stimulant effects of cocaine. In this study, we analyzed the electrophysiological effects of cocaine and A2A-Rs antagonists in striatal slices and the motor effects produced by this pharmacological modulation in rodents.

**Principal Findings:**

Concomitant administration of cocaine and A2A-Rs antagonists reduced glutamatergic synaptic transmission in striatal spiny neurons while these drugs failed to produce this effect when given in isolation. This inhibitory effect was dependent on the activation of D2-like receptors and the release of endocannabinoids since it was prevented by L-sulpiride and reduced by a CB1 receptor antagonist. Combined application of cocaine and A2A-R antagonists also reduced the firing frequency of striatal cholinergic interneurons suggesting that changes in cholinergic tone might contribute to this synaptic modulation. Finally, A2A-Rs antagonists, in the presence of a sub-threshold dose of cocaine, enhanced locomotion and, in line with the electrophysiological experiments, this enhanced activity required activation of D2-like and CB1 receptors.

**Conclusions:**

The present study provides a possible synaptic mechanism explaining how caffeine-like compounds could enhance psychomotor stimulant effects of cocaine.

## Introduction

Cocaine dependence is difficult to treat also because it is often associated with the abuse of other psychoactive compounds [1]. It causes hyperactivity and locomotor sensitization [2]–[4]. Cocaine blocks the dopamine (DA) transporter (DAT), decreases DA reuptake increasing extracellular DA levels in different brain regions, including the striatum [5]–[13]. This effect of cocaine alters glutamatergic striatal synaptic transmission [14] and affects motor function [15], [16].

Cocaine self-administration raise even more concerns when relevant doses of other commonly assumed psychoactive compounds, such as caffeine-containing beverages, are used together with this drug. In fact, caffeine is a well-known psychoactive drug displaying multiple effects on the central nervous system and specifically antagonizing adenosine receptors [17], [18]. The psychomotor stimulant effect of caffeine and its interaction with endogenous DA [19], [20] might play a significant role in the motor abnormalities induced by cocaine *via* blockade of adenosine A2A receptors located on striatal medium spiny neurons (MSNs) [18]. Although A2A-Rs have been classically located on D2-Rs expressing striato-pallidal projecting neurons [21]–[23], we have recently demonstrated that D2 and A2A-Rs are also co-expressed in striatal large aspiny (LA) cholinergic interneurons [24]. Moreover, we have postulated that the D2/A2A-R-mediated modulation of firing activity of these cholinergic interneurons might, in turn, influence the excitatory synaptic transmission in MSNs of both the direct and indirect pathways *via* the retrograde release of endocannabinoids (eCBs). Accordingly, the endocannabinoid system finely interacts with striatal glutamatergic and dopaminergic transmission [24]–[28].

Thus, the aim of the present study is to characterize the synaptic interaction between cocaine and A2A-R antagonists in distinct striatal neuronal subtypes and to explore the possibility that this interaction influences motor activity providing a possible model to explain how the concomitant use of caffeine-containing beverages exacerbates behavioral and motor alterations induced by cocaine.

## Results

### Cocaine and A2A Adenosine Receptors Antagonists Reduce Excitatory Striatal Synaptic Transmission

**Figure 1:**
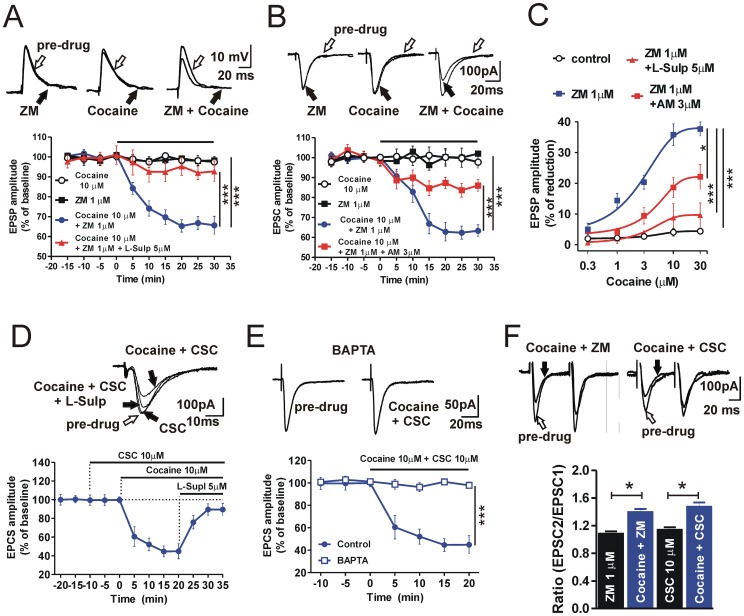
Effects of combined administration of cocaine and A2A-R antagonists on evoked EPSPs and EPSCs in striatal MSNs. (**A**) Graph of the time-course of EPSP amplitude recorded in the presence of cocaine (10 µM) or ZM241385 (ZM, 1 µM) given in isolation and cocaine plus ZM bath applied together with or without L-sulpiride (L-sulp, 5 µM), (cocaine plus ZM, *vs* cocaine F_(9,72)_ = 14.8, P<0.001; cocaine plus ZM plus L-sulp, *vs* cocaine plu ZM F_(9,72)_ = 6.6, P<0.001). Example of superimposed EPSP pairs (top traces) before and 30 min after the application of ZM, cocaine, or cocaine plus ZM. (**B**) Time-course of the EPSC amplitude in the presence of cocaine or ZM in isolation and cocaine plus ZM applied with or without AM251 (AM, 3 µM), (cocaine plus ZM, *vs* cocaine F_(9,72)_ = 16.1, P<0.001; cocaine plus ZM plus AM, *vs* cocaine plus ZM, F_(9,90)_ = 10.9, P<0.001). Example of EPSC superimposed pairs (top traces) before and 30 min after the application of ZM, cocaine, or cocaine plus ZM. (**C**) Dose-response curves of the reduction of the EPSP amplitudes induced by cocaine in control conditions and in the presence of either ZM, ZM plus AM or ZM plus L-sulp (5 µM), (cocaine plus ZM *vs* L-sulp plus cocaine plus ZM, F_(4,24)_ = 7.3, P<0.001; cocaine plus ZM plus AM *vs* cocaine plus ZM, F_(4,36)_ = 3.0, P<0.05). (**D**) Time-course showing the averaged EPSC amplitudes in the presence of 10 µM CSC, and after the subsequent application of CSC plus 10 µM cocaine. Note that the subsequent co-application of 5 µM L-sulp almost completely reverted the EPSC reduction achieved in the presence of CSC plus cocaine. EPSCs superimposed traces (top) recorded from a single striatal MSN in control condition (pre-drug), in CSC alone, in CSC plus cocaine, and in CSC plus cocaine plus L-sulp. (**E**) Time-course showing the lack of effect of cocaine plus CSC co-application on the EPSP amplitude measured in MSNs recorded either with a BAPTA-containing internal solution and with the standard solution (BAPTA internal solution, *vs* standard solution, F_(6,54)_ = 26.1, P<0.001). Example EPSC traces measured from a MSN recorded with a BAPTA-containing electrode in control condition and after the co-application of 10 µM cocaine plus 10 µM CSC. (**F**) Histogram showing the increased EPSC ratios (EPSC_2_/EPSC_1_) in the presence of cocaine plus ZM or cocaine plus CSC in respect to pre-drug condition (cocaine plus ZM *vs* pre-drug, t_(2)_ = 4.3, P<0.05; cocaine plus CSC *vs* pre-drug, t_(2)_ = 6.4, P<0.05). *P<0.05; ***P<0.001.

Sharp electrodes and whole-cell patch-clamp recordings were obtained from electrophysiologically identified MSNs from dorsal striata [29], [30]. Stimulations of glutamatergic afferents, in the presence of the GABA_A_-R antagonist BMI (10 µM), evoked EPSPs and EPSCs during intracellular and patch-clamp recordings, respectively (Figure 1). A stable response was recorded for 10–15 min and subsequently cocaine (10 µM), or A2A-R antagonists ZM241385 (ZM, 1 µM) or 8-(3-Chlorostyryl)-caffeine (CSC, 10 µM) were bath-applied in isolation. These drugs did not affect *per se* the amplitude of the postsynaptic response, on the contrary, the co-application of 10 µM cocaine plus 1 µM ZM or plus 10 µM CSC, significantly reduced the EPSPs and/or EPSCs amplitudes in respect to the baseline (Figure 1*A,B,D*). In fact, in the presence of cocaine plus ZM the EPSP amplitude was reduced by 34.3±4.5%, (n = 6, P<0.001, Figure 1*A*), and the EPSC by 36.7±6.8% (n = 6, P<0.001, Figure 1*B*), whereas in the presence of cocaine plus CSC, the EPSC amplitude was reduced by 55.1±9.3%, (n = 5, Figure 1*D*). The effect of cocaine in the presence of A2A-R blockade was dose-dependent (n = 4 for each dose, P<0.001; Figure 1*C*). Cocaine and ZM, applied in the presence of the D2 receptor antagonist L-sulpiride (5 µM), failed to reduce the EPSP amplitude (n = 4, P>0.05, Figure 1*A* and dose-response curve, n = 4 for each dose, P>0.05, Figure 1*C*). Furthermore, 15 min application of L-sulpiride almost completely reverted the synaptic inhibition caused by 20 min of cocaine and CSC co-application (n = 5, Figure 1*D*). These experiments confirmed the pivotal role of the D2 DA receptor in the D2/A2A interaction exerted by cocaine and A2A-Rs antagonists.

### Cocaine and A2A-R Dependent Reduction of Striatal Synaptic Transmission is Mediated by Postsynaptic Intracellular Calcium and Involves Presynaptic Cannabinoid Receptor 1 Activation

Striatal short-term modulation of excitatory synaptic transmission in the striatum is a response that can be modulated by the retrograde action of endogenous cannabinoids [24], [31]. In order to test whether the inhibition of excitatory synaptic transmission mediated by cocaine co-administered with A2A-R antagonists is mediated by eCBs, we co-applied cocaine and ZM in the presence of the CB1-R antagonist AM251 (3 µM). In this conditions the inhibition of the EPSC amplitude was in fact reduced only by 14±3.0% (n = 6, P<0.001, Figure 1*B*) and this effect was observed at various concentrations of cocaine (P<0.05, Figure 1*C*). Furthermore, in a subset of experiments, we patch-clamped a group of MSNs including BAPTA into the recording pipette, in order to buffer intracellular calcium levels. Under this condition, the co-application of 10 µM cocaine plus 10 µM CSC did not produce any change in the EPSC amplitude (n = 6; Figure 1*E*) confirming the pivotal role of intracellular calcium levels in the CB1-R-mediated effects of cocaine and A2A-R antagonist co-administration.

In previous studies we found that paired-pulse facilitation of the synaptic response in MSNs during the co-application of a D2-DA receptor agonist and A2A-R antagonists suggests the involvement of a presynaptic mechanism in the reduction of the excitatory transmission since this parameter was increased [24], [32]. Therefore, we tested the effect of combined cocaine and A2A-R antagonist on EPSCs evoked by paired stimuli (50 ms interval), assuming that cocaine indirectly facilitates D2-R stimulation by enhancing endogenous DA levels. Paired-pulse ratio (PPR) values in the presence of 10 µM cocaine plus the A2A-R antagonists ZM (n = 3) or CSC (n = 3) were significantly increased compared to control conditions (P<0.05; Figure 1*F*) confirming that cocaine, in combination to A2A-R antagonism, inhibits glutamatergic synaptic transmission by a presynaptic mechanism of action.

### Cocaine and A2A-R Antagonist Interaction on Excitatory Synaptic Transmission in D1-and D2-Receptor Expressing Striatal MSNs

The classical model of basal ganglia functioning suggests that the ability of DA to modulate motor control is due to the segregation of D1 and D2 DA receptors in two distinct groups of striatal MSNs. According to this model, D1 receptor (D1-R)-expressing MSNs of the direct pathway project to the *substantia nigra pars reticulata* while D2 receptor (D2-R)-expressing MSNs of the indirect pathway project to the medial *globus pallidus* [33].

**Figure 2:**
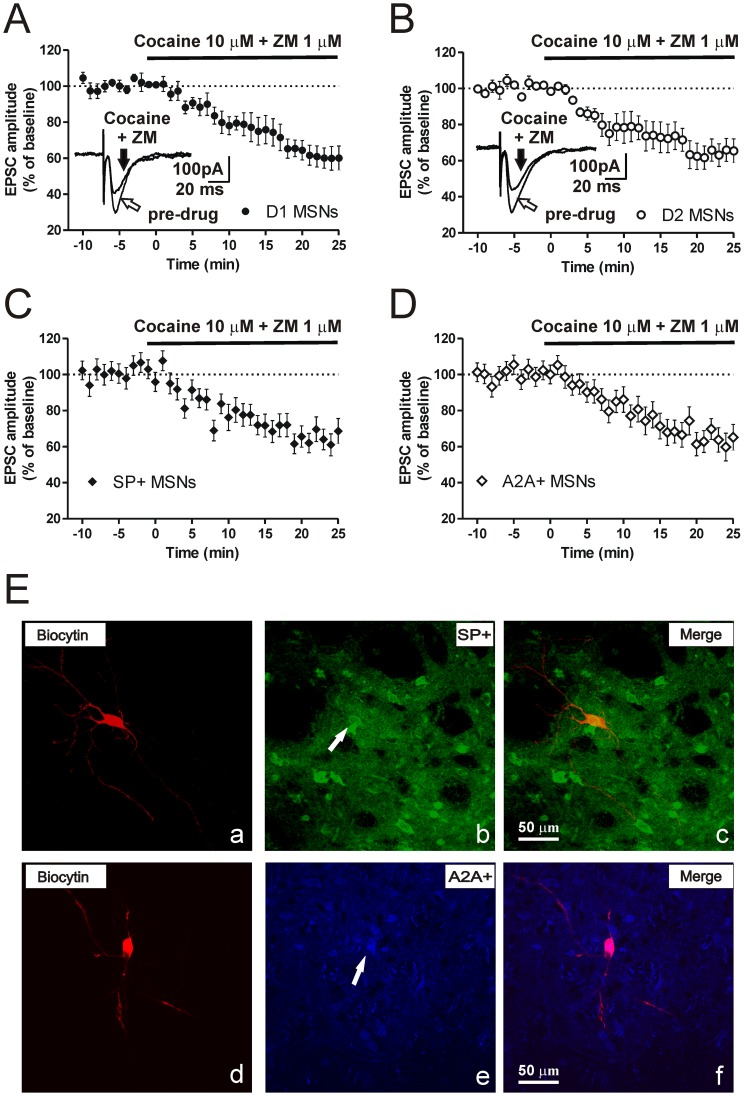
Cocaine and A2A-R pharmacological modulation affects excitatory synaptic transmission in both D2-and D1-receptor expressing striatal MSNs. Time-course plots of the EPSC amplitude and EPSC superimposed pairs recorded in the presence of cocaine plus ZM in D1-EGFP MSNs (**A**) and in D2-EGFP MSNs (**B**). Effect of cocaine plus ZM co-application on the time-course of EPSC amplitude recorded in substance P (SP) positive (**C**) and A2A positive (**D**) MSNs. (**E**) Representative images from confocal laser scanning microscopy of the immunofluorescence from a biocytin and SP containing MSN (top) and from a biocytin and A2A-R containing MSN (bottom) and the relative merged images.

Thus, we investigated whether the blockade of DAT by cocaine in conjunction to A2A-R antagonism could affect glutamatergic synaptic transmission in the entire MSN population or in a single subset of neurons. Striatal MSNs obtained from mice expressing BAC-EGFP under the control of D1-R promoter (D1-EGFP) or D2-R promoter (D2-EGFP) were visualized with an infrared and fluorescence-equipped microscope. Only neurons that displayed a marked fluorescence were approached for patch-clamp recordings and underwent subsequent standard electrophysiological characterization. We bath applied 10 µM cocaine plus 1 µM ZM, in the continuous presence of 10 µM BMI, after recording a stable EPSC baseline both in D1-EGFP- and in D2-EGFP-MSNs. In these conditions the EPSC amplitude was reduced by 39.9±6.6% in D1-EGFP-MSNs (n = 5) and by 34.5±6.8% in D2-EGFP-MSNs (n = 6), (Figure 2*A,B*).

To verify that the observed pharmacological effect induced by cocaine and A2A-R antagonists was expressed in all MSNs, in a subset of experiments, we combined electrophysiological recordings and immunohistochemical identification of the neurons. Thus, we patch-clamped MSNs using a biocytin-containing pipette in order to allow subsequent immunohistochemical identification of substance P-positive or A2A-positive MSNs, (Figure 2*E*). As shown in figure 2C and 2D, the concomitant application of 10 µM cocaine plus 1 µM ZM reduced the EPSC amplitude in SP-positive and A2A-positive MSNs by 32.0±6.9% (n = 6) and 34.8±7.2% (n = 6) respectively, providing an additional confirmation that the reduction of excitatory synaptic response exerted by cocaine in the presence of A2A-R antagonists is not segregated in a subpopulation of MSNs (Figure 2*E*).

### Cocaine and A2A Adenosine Receptors Antagonists Reduce the Frequency of Glutamate Spontaneous Release in Striatal MSNs by a CB1 Receptor-mediated Action

**Figure 3:**
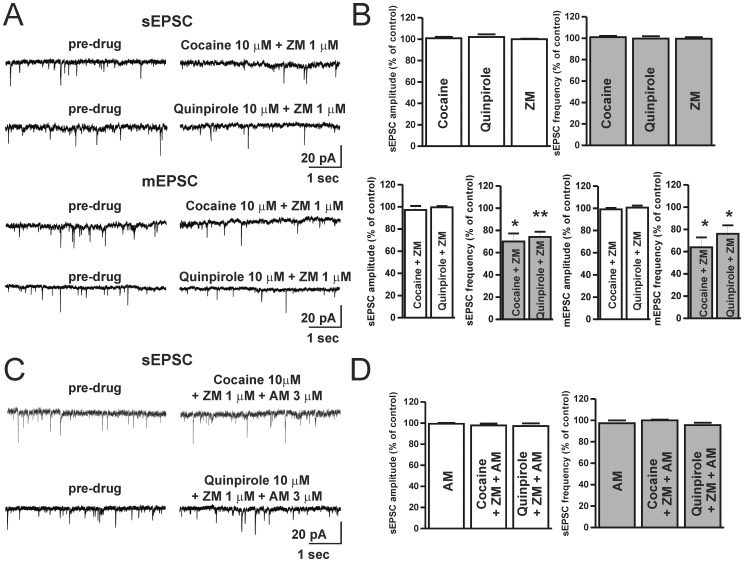
The co-application of cocaine or quinpirole together with A2A-R antagonist similarly reduce spontaneous EPSC by a CB1 receptor-dependent action. (**A**) Representative traces of sEPSCs and mEPSCs recorded in control conditions (pre-drug) and in the presence of 10 µM cocaine plus 1 µM ZM or 10 µM quinpirole plus 1 µM ZM. (**B**) Histogram of averaged amplitude and frequency of sEPSCs in the presence of cocaine, quinpirole and ZM applied in isolation (top). Averaged amplitude and frequency of sEPSCs and mEPSCs measured in the presence of cocaine or quinpirole co-applied with ZM (bottom), (sEPSC frequency: cocaine plus ZM, *vs* pre-drug, t_(5)_ = 3.7, P<0.05; cocaine plus quinpirole, *vs* pre-drug, t_(7)_ = 4.6, P<0.01. mEPSC frequency: cocaine plus ZM, *vs* pre-drug, t_(4)_ = 3.2, P<0.05; cocaine plus quinpirole, *vs* pre-drug, t_(4)_ = 2.9, P<0.01). (**C**) Traces of sEPSCc in control conditions (pre-drug) and after the co-application of cocaine or quinpirole plus ZM and the CB1-R antagonist AM251. (**D**) Averaged sEPSC amplitude and frequency in the presence of AM251, cocaine plus ZM plus AM or quinpirole plus ZM plus AM. *P<0.05; **P<0.01.

We investigated the possible role of the combined activation of D2-Rs and the inhibition of A2A-Rs by measuring the spontaneous synaptic activity of excitatory inputs to striatal MSNs. We stimulated D2-Rs, either by applying cocaine, that enhances endogenous DA levels, or by applying the selective D2-R agonist quinpirole. SEPSCs were recorded in the continuous presence of 10 µM BMI to exclude GABAergic inhibitory components of synaptic activity. In these conditions (pre-drug), the amplitude and the frequency of sEPSCs were firstly recorded in the presence of cocaine (10 µM, n = 4), D2-R agonist quinpirole (10 µM, n = 6), or A2A-R inhibitor ZM241385 (1 µM, n = 7). These drugs did not affect *per se* the spontaneous release of excitatory neurotransmitter, in fact, neither the amplitude nor the frequency of sEPSCs were different from that measured in pre-drug conditions (P>0.05, Figure 3*B*). On the contrary, while the combined application of cocaine plus ZM or quinpirole plus ZM did not affect the sEPSC amplitude, the frequency was significantly affected (Figure 3*A,B*). In fact, the sEPSC frequency in the presence of cocaine plus ZM was reduced by 28.6±19% (n = 6, P<0.05) and in the presence of quinpirole plus ZM by 27.0±5.6% (n = 8, P<0.01) in respect to the relative pre-drug condition (Figure 3*A,B*). Furthermore, we recorded spontaneous EPSCs in the continuous presence of BMI plus 1 µM TTX in order to measure miniature EPSCs (mEPSCs), representing action potential-independent spontaneous release of neurotransmitter. Also in this case, while mEPCSs amplitude was not affected either in the presence of 10 µM cocaine plus 1 µM ZM (P>0.05) or in the presence of 10 µM quinpirole plus 1 µM ZM (P>0.05), its frequency was reduced by 38.8±6.5% (n = 5, P<0.05) in the presence of cocaine plus ZM and by 25.5±7.4% (n = 5, P<0.05) in the presence of quinpirole plus ZM, relatively to the respective pre-drug condition (Figure 3*A,B*).

As observed for the modulation of the evoked synaptic transmission, when the experiment was performed in the presence of the CB1-R antagonist AM251 (3 µM), the reduction of the frequency of spontaneous EPSC in the presence of cocaine or quinpirole, co-applied with the A2A-R antagonist ZM, did not occur (cocaine plus ZM plus AM *vs* AM, n = 5, P>0.05; quinpirole plus ZM plus AM *vs* AM, n = 5, P>0.05, Figure 3*C,D*).

### Cocaine and A2A Receptor-mediated Regulation of Firing Activity in Striatal Cholinergic Interneurons

We recently reported that choline acetyl transferase (ChAT)-positive striatal large aspiny (LA) interneurons co-express D2- and A2A-Rs and are deeply involved in the D2/A2A-R-dependent modulation of striatal excitatory synaptic transmission since D2-R agonist and A2A-R antagonist reduced the firing rate of these interneurons that potentially control the synaptic transmission of the entire population of striatal MSNs [24]. Cocaine administration might therefore also exert a similar action on D2-Rs expressed on LA interneurons by its direct effect on the DAT, thus increasing intrastriatal DA levels.

**Figure 4:**
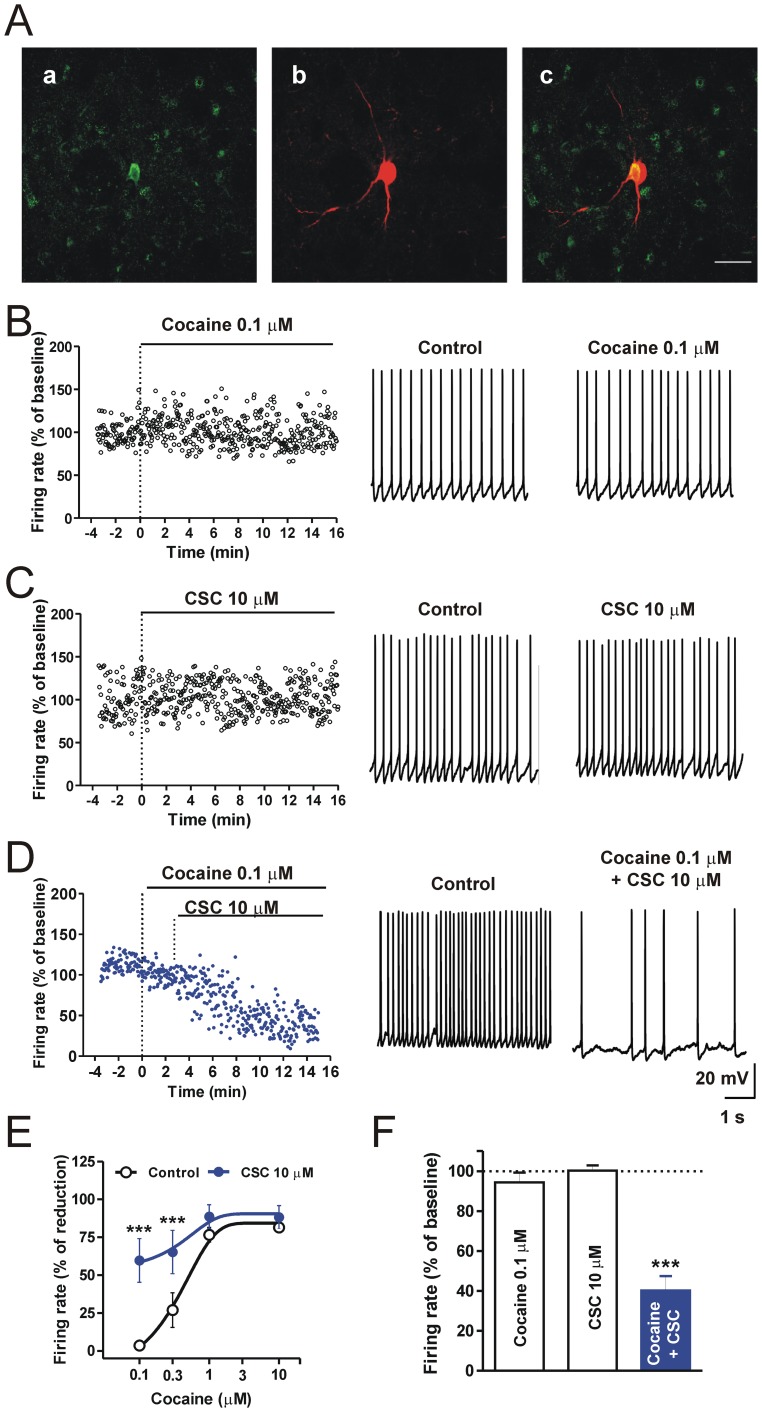
Cocaine and 8-(3-Chlorostyryl)-caffeine co-application reduce the firing discharge rate of striatal cholinergic interneurons. (**A**) Representative confocal laser scanning microscopy images of striatal cholinergic interneurons filled with biocytin during electrophysiological recordings. Double immunofluorescence detection of biocytin and anti-choline acetyl transferase (ChAT). Biocytin is visualized in green-cy2 fluorescence (a), ChAT in red-cy3 fluorescence (b). Left panel is the merged image (c) Scale bar: 50 µm. (**B**) Representative time-course of the firing rate and example of trace recordings from cholinergic interneurons in control conditions and in the presence of 0.1 µM cocaine (**B**), 10 µM CSC (**C**) or cocaine plus CSC (**D**), respectively. Plotted mean frequency of the firing activity is calculated in time windows of 5 seconds. (**E**) Dose-response curves of spike frequency from cholinergic interneurons measured in the presence of cocaine or cocaine plus CSC (cocaine, *vs* cocaine plus CSC, F_(3,28)_ = 6.5, P<0.01). (**F**) Histogram of averaged firing frequency of cholinergic interneurons recorded in the presence of cocaine, CSC and cocaine plus CSC, cocaine vs cocaine plus CSC, Bonferroni’s *post-hoc* test, ***P<0.001.

LA interneurons were patch-clamped in current-clamp mode and, as shown in figure 4*A,B,C,E*, neither a low dose of cocaine (0.1 µM, n = 5) nor 10 µM of the caffeine-derivative CSC (n = 4) affected either the resting membrane potential or the firing rate of these neurons when applied in isolation. However, cocaine reduced the firing frequency of these neurons in a dose-dependent manner at higher concentrations (n = 5 for each dose, Figure 4*E*). The co-application of cocaine plus 10 µM CSC significantly enhanced cocaine effect of the firing frequency (P<0.001). In fact, in the presence of CSC, even 0.1 µM cocaine was able to reduce the neuronal firing frequency of LA interneurons by 59.7±7.2% (Figure 4*D,E,F*).

The block of DA transporter and the subsequent increase of endogenous DA levels together with A2A adenosine signaling might thus regulate the firing discharge of cholinergic interneurons toward MSNs and subsequently regulate striatal excitatory synaptic transmission by an eCB-mediated mechanism [24].

### Activation of M1-Like Muscarinic Receptor is Required for the Reduction of Excitatory Synaptic Transmission Induced by Cocaine and A2A-R Antagonist

**Figure 5:**
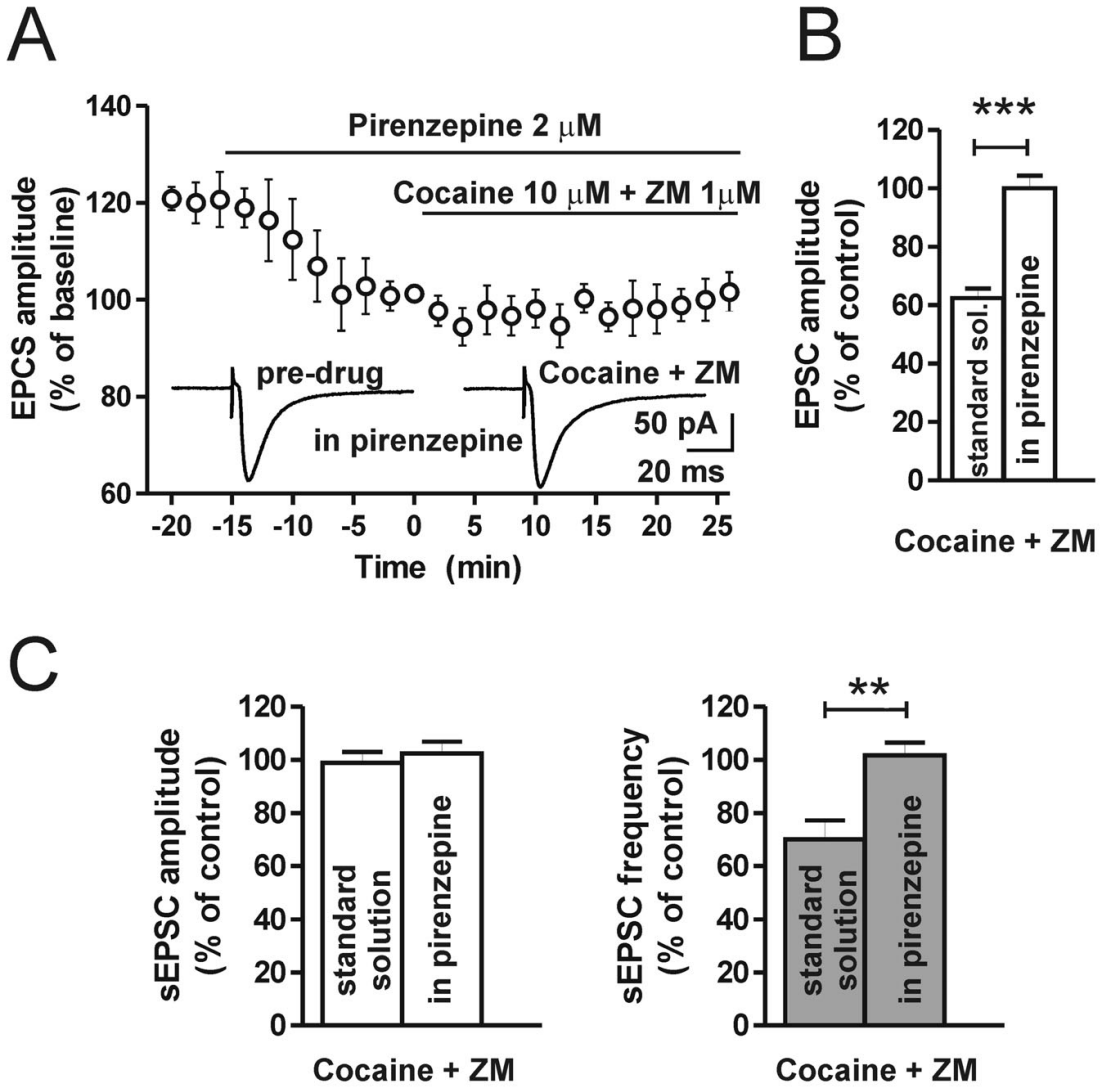
M1-like muscarinic receptor blockade prevents the reduction of excitatory synaptic transmission induced by cocaine and A2A-R antagonist. (**A**) Time-course showing evoked EPSC amplitude recorded from striatal MSNs in the continuous presence of the M1-like receptor blocker pirenzepine (2 µM). 10 µM cocaine plus 1 µM ZM are bath applied during pirenzepine perfusion. Representative traces of EPSC acquired 2 minutes before (left) and 20 minutes after (right) the co-application of cocaine plus ZM, bath applied 15 minutes after the onset of pirenzepine. (**B**) Histogram showing the effect on the EPSC amplitude of 25 minutes co-application of cocaine plus ZM in the presence of pirenzepine compared to the effect in the standard solution (EPSC amplitude t_(10)_ = 6.9, P<0.001). (**C**) Histograms showing the effect of co-application of cocaine plus ZM on spontaneous EPSC amplitude (left) and frequency (right), in the presence of pirenzepine or in comparison to the effect in the standard solution (sEPSC frequency t_(12)_ = 3.9, P<0.01). **P<0.01; ***P<0.001.

In physiological conditions D2-R agonists, in combination with A2A-Rs antagonists, were able to reduce striatal cholinergic transmission and, in turn to inhibit glutamate release toward MSNs acting on postsynaptic M1-like muscarinic receptors [24]. In order to verify the involvement of M1-like receptors in the cocaine-and A2A-R-mediated reduction of striatal glutamatergic transmission we recorded evoked and spontaneous MSNs EPSCs in the continuous presence of 2 µM pirenzepine applied for the whole duration of the experiments. After 15 minutes in pirenzepine (pre-drug), bath application of 10 µM cocaine plus 1 µM ZM for 25 minutes did not produce any change of the evoked EPSCs (n = 6; P>0.05; Figure 5*A,B*). Spontaneous EPSCs were also recorded after 15 minutes of pirenzepine (pre-drug) and then after 20–25 minutes of cocaine and ZM application. In these conditions the sEPSCs were also not affected, in fact, neither the sEPSC amplitude nor the frequency were altered by cocaine plus ZM application in respect to pre-drug conditions (n = 8; P>0.05; Figure 5*C*). Thus, blockade of M1-like receptors occluded cocaine and A2A-R mediated reduction of glutamatergic transmission (P<0.01; Figure 5*B,C*).

### Motor Stimulation Induced by Cocaine and A2AR Antagonists Co-administration is Influenced by CB1 and D2 Receptors

To unveil if reduced glutamatergic synaptic excitatory transmission in both D2- and D1-R-expressing MSNs, produced by concomitant exposure to cocaine and A2A-Rs antagonists, might have direct behavioral consequences, we analyzed motor effects produced by the co-administration of these drugs.

**Figure 6:**
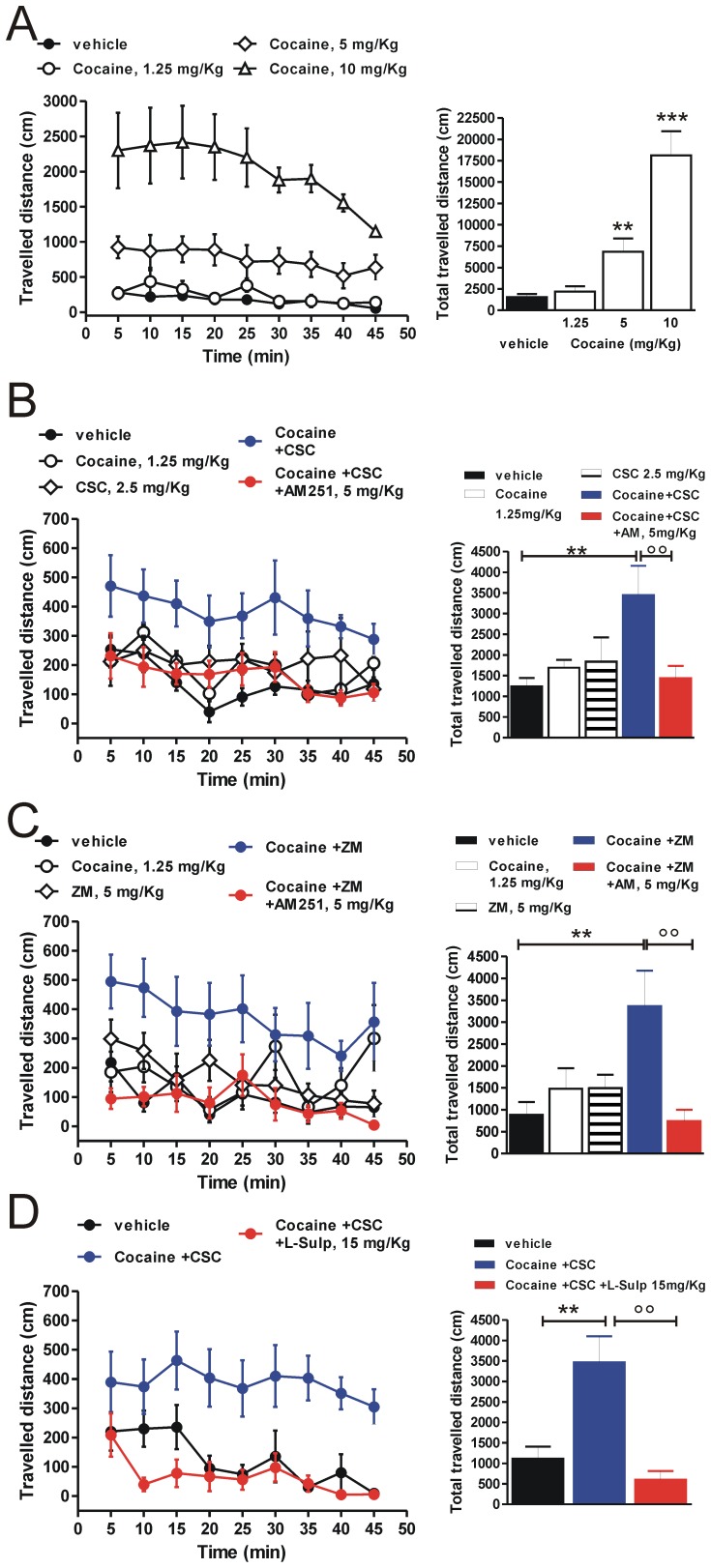
Influence of CB1 and D2 receptors on motor activity induced by co-administration of cocaine and A2A-R antagonists. (**A**) Effect of 1.25, 5 and 10 mg/kg cocaine on locomotor activity of C57BL/6 mice (n = 12 vehicle, n = 6 per each dose of cocaine). (**B**) Locomotor activity induced by administration of vehicle (n = 6), 1.25 mg/kg cocaine (n = 6), 2.5 mg/kg CSC (n = 6), cocaine-CSC co-administration (n = 6), cocaine-CSC plus 5 mg/kg AM251 injection (n = 12). (**C**) Locomotor activity induced by administration of vehicle (n = 9), 1.25 mg/kg cocaine (n = 6), 5 mg/kg ZM241385 (n = 10), cocaine-ZM241385 co-administration (n = 6), cocaine-ZM241385 plus 5 mg/kg AM251 injection (n = 6). (**D**) Locomotor activity induced by vehicle (n = 6), cocaine-CSC co-administration (n = 7) and cocaine-CSC plus 15 mg/kg L-sulpiride injection (n = 6). Locomotor activity is shown as both time course (left panels) and total distance travelled (right panels) over 45-min test sessions. All values are expressed as mean ± SEM. **P<0.01, compared to vehicle-treated mice; °°P<0.01, compared to mice co-administered with cocaine and A2A-R antagonists (CSC or ZM241385), (Fisher’s *post-hoc* analyses).

First, we evaluated motor response of mice to different doses of cocaine (1.25, 5 or 10 mg/kg) in order to find the concentration that was ineffective to stimulate locomotor activity (Figure 6*A*). Among the doses tested, 1.25 mg/kg did not show significant effects on motor stimulation, compared to vehicle treatment (P>0.05). Therefore, 1.25 mg/kg cocaine was chosen as the sub-effective dose for the co-administration experiments described below.

Secondary, we analyzed the effect of concomitant administration of cocaine and CSC, a selective A2A-R antagonist (Figure 6*B*). Before co-administration, we also tested the sub-effective dose of CSC, that resulted in 2.5 mg/kg (data not shown). We found that co-administration of ineffective doses of cocaine and CSC was able to induce a significant increase of motor response, compared with injections of vehicle, cocaine or CSC alone (P<0.01, per each treatment; *post-hoc* analysis). Interestingly, treatment with the selective CB1-R antagonist AM251 (5 mg/kg) abolished the motor stimulation induced by co-administration of cocaine and CSC (P<0.01, *post-hoc* analysis). To confirm this observation, we tested another selective A2A-R antagonist, such as ZM241385 (Figure 6*C*). A pilot test indicated as 5 mg/kg the sub-effective dose of ZM241385 for co-administration experiment (data not shown). Similarly to CSC, mice co-administered with ineffective doses of cocaine and ZM241385 showed a significant increase of locomotion, compared to mice treated with vehicle, cocaine or ZM241385 alone (P<0.01, per each treatment). Also in this case, motor activity induced by co-administration was suppressed when mice received also AM251 (P<0.01).

Electrophysiological analysis indicated that, besides CB1-R activation, stimulation of D2-Rs plays as well a main role in modulating the striatal excitatory synaptic transmission. Therefore, we investigated the effect of the D2-R antagonist L-sulpiride on motor activity induced by cocaine-CSC co-administration (Figure 6*D*). Statistical analysis showed that treatment with 15 mg/kg L-sulpiride was able to inhibit the motor hyperactivity evoked by concomitant injection of cocaine and CSC (P<0.01), thus confirming electrophysiological findings.

## Discussion

In this work we examined the effects of cocaine on striatal excitatory synaptic transmission and motor activity when administered in combination with A2A adenosine receptor antagonists. Given the physiological and social impact of assumption of cocaine in the presence of other psychotropic drugs, such as caffeine-like compounds, that specifically target endogenous adenosinergic signaling, to block A2A-Rs we used the caffeine-derivative 8-(3-chlorostyryl)-caffeine and ZM241385. In this study we provided for the first time electrophysiological evidence of a significant inhibition of striatal excitatory synaptic transmission exerted by cocaine in combination with A2A-R antagonists. In a previous our publication [24], we have already reported that the concomitant activation of D2 receptors by “exogenous” agonists and the blockade of A2A receptors reduce striatal glutamatergic transmission both in physiological condition and in experimental models of Parkinson’s disease. Here, however, we describe for the first time the striatal electrophysiological effects produced by the cocaine-induced increase of “endogenous dopamine” and its interaction with A2A receptors. Moreover, we have also addressed the possible behavioural implication of this interaction on motor activity. We demonstrated that the effect of cocaine in this interaction is produced by the activation of D2 DA-R, since L-sulpiride prevents the reduction of the excitatory postsynaptic response in MSNs. The importance of DA signaling, involving the activation of D2-Rs, is strengthened by the analysis of MSNs spontaneous excitatory events. In fact, we found that the frequency, but not the amplitude, of spontaneous EPSCs was only reduced by the combined superfusion of cocaine or the D2-R agonist quinpirole plus an A2A-R antagonist. The effect on the frequency but not on the amplitude, suggests the involvement of a presynaptic mechanism activated by these drugs. Therefore, increased striatal DA level, achieved by cocaine-induce DAT blockade, appears to be a pivotal signal required for the reduction of cocaine and A2A-R-mediated glutamatergic neurotransmission.

Cocaine and A2A-R inhibition of synaptic response also required postsynaptic intracellular calcium changes and the activation of CB1-Rs since intracellular BAPTA or bath application of a CB1-R antagonist significantly opposed this inhibitory response. Thus, according to previous published works [25], [27], [32], [34], [35], eCBs released by postsynaptic MSNs by a calcium-mediated mechanism, reduce glutamatergic striatal neurotransmission by stimulating presynaptic CB1-Rs. Our analysis of the changes of paired-pulse stimulated EPSCs revealed that cocaine and A2A-R antagonists co-application increased the PPR in MSNs, also supporting the involvement of a presynaptic mechanism of action. Furthermore, the CB1-R antagonist AM251 prevented the reduction of spontaneous EPCS frequency of MSNs in the presence of either cocaine or the D2-R agonist quinpirole, when applied in combination with a A2A-R antagonist. Considering the abundant presence of striatal CB1-Rs on presynaptic glutamatergic terminals, we provide additional evidence of a presynaptic mechanism involvement. These lines of evidence strongly support a D2-and A2A-R mediated calcium-dependent release of eCBs from postsynaptic MSNs, triggered by the combined application of cocaine and A2A-R antagonists.

Interestingly, as revealed by immunofluorescence analysis of patch-clamped MSNs from BAC-EGFP mice and from MSNs expressing substance P or A2A-Rs we found that the combined effect of cocaine and A2A-R antagonism was not segregated to specific neuronal striatal subpopulations. In fact, we report that in the presence of cocaine plus the A2A-R antagonist ZM, the inhibition of the EPSC amplitude was similar in D1-EGFP- and SP-expressing neurons as well as in D2-EGFP and A2A-R-expressing cells, identified as D1-R or D2-R expressing MSNs, respectively. According to these data it has been shown that in most of the recordings from striatal MSNs, activation of D2-Rs inhibits striatal glutamate release by a retrograde eCB signaling [36] and that cocaine can increase the level of the eCB anandamide in the striatum through the stimulation of DA D2-like receptors [37].

These findings are in line with results from our previous work in which we have shown that LA interneurons of the intrastriatal network may exert a major modulatory role of the synaptic excitatory response of both D2-R- and D1-R-expressing MSNs. This action was achieved by regulating intracellular calcium via M1 muscarinic receptors, and we showed that cholinergic firing tone was reduced by a the D2-R agonist quinpirole and a A2A-R antagonist [24], [38]. In the present work, we extended these findings providing evidence that cocaine can affect the firing frequency of LA interneurons, in a dose-dependent way, similarly to the action of D2-R agonist [24]. These findings are supported by previous studies in which increased DA release results in decreased acetylcholine release in dorsal striatum [24], [39], [40], by the presence of DA D2-Rs [41] and adenosine receptor mRNAs, including A2A-R mRNA in striatal LA interneuron, suggesting the possibility that these receptors, together with D2-R, might modulate acetylcholine release [42].

Furthermore, we found that a sub-threshold dose of cocaine that does not affect the firing frequency of LA neurons, is sufficient to produce a robust effect in reducing the firing frequency when applied in combination with the caffeine derivative CSC that specifically blocks A2A-Rs. These findings support the important role of intrastriatal cholinergic in the regulation of motor functions.

**Figure 7:**
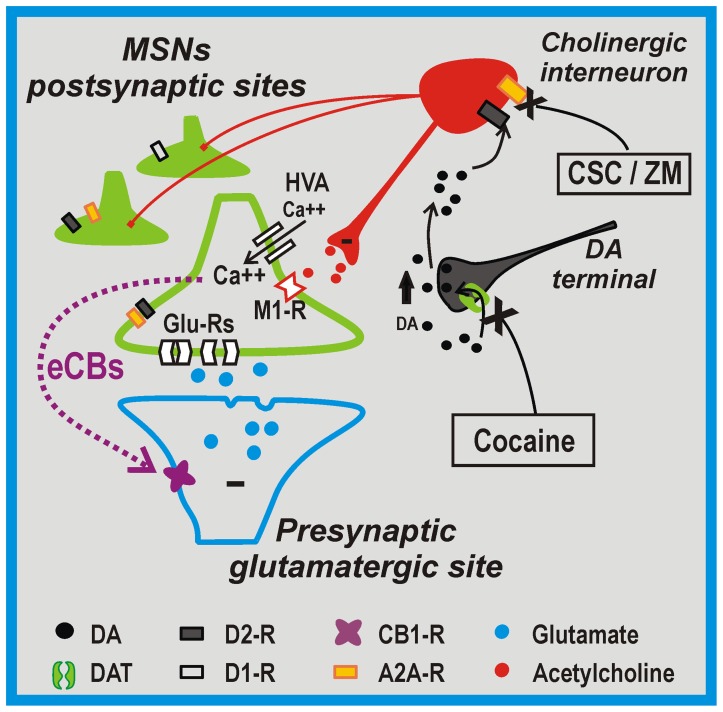
Model of intrastriatal network during cocaine and A2A-R antagonists exposure. The administration of cocaine enhances intrastriatal dopamine (DA) levels by blocking the DA transporter (DAT) expressed on DA terminals. The simultaneous effect of the stimulation of D2-Rs by DA and the blockade of A2A-Rs by CSC or ZM, on cholinergic interneurons might decrease the release of acetylcholine at the synaptic sites of D2-and D1-R expressing MSNs. The subsequent reduced stimulation of M1-like cholinergic receptors and opening of high-voltage activated Ca^2+^ channels (HVA) would trigger Ca^2+^-mediated release of endocannabinoids. This retrograde messenger might ultimately inhibit excitatory striatal excitatory transmission by the stimulation of CB1 receptors located on presynaptic glutamatergic sites.

We can hypothesize that the synergistic action of cocaine and A2A-R antagonists in inhibiting the firing rate of cholinergic interneurons would result in the reduction of endogenous acetylcholine release and the consequent reduced activation of M1 muscarinic receptors on MSNs. The established effect of M1 receptor inhibition would be the opening of L-type Ca^2+^ channels [38]. This latter event might trigger postsynaptic effects on MSNs leading to eCBs release and reduction of glutamatergic transmission by the activation of presynaptic CB1 receptors (Figure 7). Accordingly, we found that in the presence of pirenzepine, a M1 receptor inhibitor, the electrophysiological effects of cocaine plus A2A-R antagonists modulation on glutamatergic transmission were fully occluded. LA interneurons can also modulate the activity of MSNs by decreasing glutamate release via stimulation of presynaptic M2 receptors located on MSN glutamatergic afferents [43]. Presynaptic M2 receptors might therefore be involved in the fine regulation of MNS excitability together with eCBs after the concomitant application of cocaine and A2A-R antagonists.

Electrophysiological effects of combined exposure to cocaine with either CSC or ZM reflected in changes of the motor function. In fact, while cocaine administration per se increased spontaneous motor activity, as previously reported [4] in this work we characterized for the first time the additive effect of a sub-threshold doses of co-administered cocaine and A2A-R antagonists on motor activity. While previous findings have shown that the pharmacological inhibition of DA activity blocked cocaine-activated spontaneous behavior [44], our data clearly demonstrate that cocaine- and A2A-R-activated effects on mice locomotion is specifically mediated by DA D2-Rs since L-sulpiride fully prevented the motor effect observed in the presence of cocaine and CSC.

It has been already suggested that eCBs signaling mediates psychomotor activation by A2A-R antagonists [45]. The authors claimed that their finding was consistent with a model in which eCBs-mediated inhibition of the indirect pathway increases movement. This interpretation is in line with the classical theory of the segregation within the basal ganglia postulating that increased direct-pathway activity facilitates movement, while increased indirect-pathway activity inhibits movement [46], [47]. Our findings show that cocaine (via D2-R) and A2A-R antagonists control the striatal cholinergic system and subsequently regulate eCBs production in MSNs of both direct- and indirect pathway, opening a novel and different scenario. This view, also supported by our recent electrophysiological findings in experimental models of Parkinson’s disease [24], [48], suggests that plasticity of glutamatergic transmission, rather than being expressed in distinct and opposite manner in the two pathways, affects the two striatal population subtypes leading to motor activation. In line with a previous hypothesis [38], the cholinergic interneurons seem to play a central role in orchestrating these physiological and pharmacological responses. However, the behavioural effects observed after the co-administration of cocaine and/or A2A antagonists might be under the control of different brain regions, thus alternative explanations should be taken into account. CB1-R stimulation in the ventral tegmental area, in fact, can increase the firing rate of DA neurons [49]. Furthermore, CB1-Rs activation might also contribute to increase striatal DA levels following systemic cocaine administration [50]. Therefore, the role of CB1-R activation upstream of the striatum on cocaine-evoked DA release, presumably contributes to the locomotor effects of cocaine that are inhibited by CB1-R antagonism.

## Materials and Methods

### Electrophysiology

All the experiments were conducted in conformity with the European Communities Council Directive of November 1986 (86/609/ECC) in accordance with a protocol approved by the Animal Care and Use Committee at the University of Perugia and every effort was made to minimize animal suffering. Two to three months-old male Wistar rats (Harlan) and 5–6 weeks old male C57Bl/6J mice carrying BAC that express enhanced green fluorescent protein (BAC-EGFP) under the control of D1-R promoter (*drd1a*-EGFP) or D2-R promoter (*drd2*-EGFP) were used for electrophysiological experiments. BAC-EGFP mice were originally generated by the GENSAT (Gene Expression Nervous System Atlas) program at the Rockefeller University [51].

Coronal slices including the cortex and the striatum were cut from rats (thickness, 270 µm) or from BAC-EGFP mice (thickness, 220–240 µm) brain using a vibratome. A single slice was transferred to a recording chamber and submerged in a continuously flowing Kreb’s solution (34°C; 2.5–3 ml/min) bubbled with a 95% O_2_–5% CO_2_ gas mixture. The composition of the Kreb’s solution was (in mM) 126 NaCl, 2.5 KCl, 1.2 MgCl_2_, 1.2 NaH_2_PO_4_, 2.4 CaCl_2_, 10 glucose, and 25 NaHCO_3_. Drugs were bath-applied by switching the solution to one containing known concentrations of drugs. Powders were dissolved in water or DMSO and aliquoted. Drugs were applied by dissolving them to the desired final concentration in the external Kreb’s solution [24], [52]. For intracellular recordings, electrodes were pulled from borosilicate glass pipettes, backfilled with 2 M KCl (30–60 MΩ). Only neurons electrophysiologically identified as spiny neurons were considered for experiments with sharp microelectrodes. An Axoclamp 2B amplifier (Molecular Devices) was connected in parallel to an oscilloscope to monitor the signal in “bridge” mode and to a PC for acquisition of the traces using pClamp 10 software (Molecular Devices) [29]. MSNs from slices of mice expressing BAC-EGFP under the control of D1-R promoter (D1-EGFP) or D2-R promoter (D2-EGFP) were visualized with an infrared- and fluorescence-equipped microscope (Olympus). For patch-clamp recordings, signals were amplified with a Multiclamp 700B amplifier (Molecular Devices), recorded and stored on PC using pClamp 10. Whole-cell access resistance was 5–30 MΩ, holding current ranging between 80 to −50 pA.

Whole-cell voltage-clamp recordings (holding potential, −80 mV) were performed with borosilicate pipettes (4–7 MΩ) filled with a standard internal solution containing (in mM): 145 K^+^-gluconate, 0.1 CaCl_2_, 2 MgCl_2_, 0.1 EGTA, 10 HEPES, 0.3 Na-GTP and 2 Mg-ATP, adjusted to pH 7.3 with KOH. When a BAPTA-containing internal solution was used, 20 mM BAPTA was added to the standard solution and K^+^-gluconate was lowered to 125 mM. Some electrodes were filled with the standard solution plus biocytin.

A bipolar electrode, connected to a stimulation unit (Grass Telefactor), was located in the white matter between cortex and striatum to stimulate glutamatergic fibers (0.1 Hz) and evoke excitatory postsynaptic potentials (EPSPs) and currents (EPSCs). Current traces for spontaneous EPSCs were recorded in the continuous presence of 10 µM BMI (sEPSCs) or BMI plus 1 µM tetrodotoxin to isolate miniature EPSCs (mEPSCs). Five minute episodes were acquired during the pre-drug condition and 20 minutes after of drug application. Cholinergic large aspiny (LA) interneurons, were localized under IR-DIC visualization by their large soma and subsequently identified by their electrophysiological properties. They were recorded in whole-cell current-clamp mode using an internal solution containing (in mM): 120 K^+^-gluconate, 0.1 CaCl_2_, 2 MgCl_2_, 1 EGTA, 10 HEPES, 0.3 Na-GTP and 2 Mg-ATP, adjusted to pH 7.3 with KOH. These cells presented a pronounced h-current (Ih) and a typical sag potential in response to hyperpolarizing steps of current [24], [53]–[55]. The majority of LA interneurons were firing spontaneously [54]. Cells that were not spontaneously active were injected with 10–50 pA of positive current for reaching the threshold of action potential.

Quantitative data are expressed as a percentage of EPSP and EPSC amplitudes or firing frequency with respect to the relative control values, the latter representing the mean of responses recorded during a stable period (10–15 min). The spontaneous events were detected using Clampfit 10 (Molecular Devices) and confirmed visually for accuracy. Amplitude and frequency were measured in epochs of 5 minutes.

### Tissue Processing and Double Immunofluorescence

Brain slices that were used for electrophysiological recordings were processed to verify the presence of cells filled with biocytin. Thus, they where post-fixed overnight at +4°C with 4% paraformaldehyde in saline solution and then cryo-protected in phosphate buffer (PB 0.1 M) with sodium azide 0.02% for 48 hours at 4°C. Sections were incubated with streptavidin-Cy3 diluted 1∶600 in PB-TX-100 0.3% for 2 hours at room temperature and quickly observed, under an epi-illumination ﬂuorescence microscope (Zeiss Axioskop 2).

A first set of brain slices was preincubated with a primary antibody guinea pig anti-SP or rabbit polyclonal anti-A2A to label MSNs of the “direct” or “indirect” basal ganglia pathway, respectively [23], [56]–[59]. The primary antisera were used at a concentration of 1∶400 for SP and 1∶250 for A2A in 0.1 M PB containing Triton X-100, 0.3% and sodium azide, 0.02% for 24 hours at room temperature and 48 hours at 4°C. Sections were then rinsed three times for 15 minutes at room temperature, and subsequently incubated with Alexa fluor 488 goat anti-guina pig or Alexa fluor 647 chicken anti-rabbit secondary antibody for 2 hours at room temperature. All secondary antibodies were used at 1∶200 concentration. Tissue was mounted on gelatin-coated slides, coverslipped with GEL-MOUNT and examined under an epi-illumination ﬂuorescence microscope (Zeiss Axioskop 2), and a CLSM (Zeiss LSM 700) was used to acquire all the images.

In order to label biocytin-filled cholinergic interneurons, a second set of slices, used for electrophysiological recordings, was also processed to detect the presence of choline acetyl transferase. Slices were incubated with a primary antibody rabbit anti-choline acetyl transferase (ChAT; Immunological Science, Rome Italy) to label the cholinergic interneurons. The primary antisera were used at a 1∶400 concentration in 0.1 M PB containing Triton X-100, 0.3% and sodium azide, 0.02% for 24 h a room temperature and 48 h at 4°C. Sections were then rinsed three times for 15 min at room temperature, and subsequently incubated with a goat anti-rabbit Cy2-conjugated secondary antibody (Jackson Immunoresearch, West Grove, PA, USA) for 2 hours at room temperature. Tissue was mounted on gelatin-coated slides, coverslipped with GEL-MOUNT and examined under an epi-illumination fluorescence microscope (Zeiss Axioskop 2), and a CLSM (Zeiss LSM 510) was used to acquire all the images.

### Motor Responses Induced by Drugs

Locomotor activity was analyzed as previously described [60]. To investigate the effect of CB1-R-dependent stimulation on locomotor activity induced by cocaine and the A2A-R antagonist, CSC, C57BL/6 mice were divided in 5 groups, each of them receiving: (1) vehicle, (2) cocaine alone (1.25 mg/kg), (3) CSC alone (2.5 mg/kg), (4) cocaine plus CSC, (5) cocaine plus CSC plus AM 251 (5 mg/kg). Similarly, to investigate the effect of CB1-R stimulation on locomotor activity induced by cocaine and the A2A-R antagonist, ZM241385, mice were divided in 5 groups, each of them receiving: (1) vehicle, (2) cocaine alone (1.25 mg/kg), (3) ZM241385 alone (5 mg/kg), (4) cocaine plus ZM241385, (5) cocaine plus ZM241385 plus AM 251 (5 mg/kg). Finally, to study the effect of D2-R stimulation on locomotor activity induced by cocaine and A2A-R antagonist CSC, mice were divided in 3 groups, each of them receiving: (1) vehicle, (2) cocaine plus CSC, (3) cocaine plus CSC plus L-sulpiride (15 mg/kg). Drugs or vehicle were injected intraperitoneally.

Mice were first habituated to the experimental cage for 60 min and then injected with the drugs reported above. CSC, AM251, ZM241385 and L-sulpiride were dissolved in vehicle (50% polyethylene glycol, PEG and 50% saline) whereas cocaine was prepared in saline. Recording of locomotor activity started 15 min after last injection and was detected over test session 45 min by a computerized video tracking system (Videotrack, Viewpoint S.A. Champagne au Mont d’Or, France). Total distance traveled was analyzed by ANOVA, followed by Fisher’s *post-hoc* analysis, when required.

### Statistical Analysis

Off-line analysis was performed using Clampfit 10 (Molecular Devices) and GraphPad Prism 5.0 (GraphPad Software Inc.) software. Two-way ANOVA or Student’s *t*-test were used. Values are mean ± SE; “n” is the number of recorded neurons. The significance level was established at P<0.05 (*), P<0.01 (**) and P<0.001 (***).

### Chemicals

AM251 (AM), pirenzepine dihydrochloride, (-)-quinpirole hydrocholoride, (L)-sulpiride, tetrodotoxin citrate (TTX), ZM241385 (ZM) were from Tocris-Cookson; (-)-bicuculline methiodide (BMI), cocaine, 8-(3-Chlorostyryl)-caffeine (CSC), streptavidin-Cy3 were from Sigma-Aldrich. Anti-SP was from abcam; anti-A2A was from Alexis; alexa fluor 488 and alexa fluor 647 were from Invitrogen, Molecular probes.
